# ﻿Three new species of the genus *Gamasomorpha* Karsch, 1881 (Araneae, Oonopidae) from the Philippines and Sumatra, Indonesia

**DOI:** 10.3897/zookeys.1258.168616

**Published:** 2025-11-06

**Authors:** Yang Bai, Dongju Bian, Yanfeng Tong, Christoph Hörweg

**Affiliations:** 1 College of Life Science, Shenyang Normal University, Shenyang 110034, Liaoning, China Shenyang Normal University Shenyang China; 2 Institute of Applied Ecology, Chinese Academy of Sciences, Shenyang 110016, China Institute of Applied Ecology, Chinese Academy of Sciences Shenyang China; 3 3. Zoology, Natural History Museum Vienna, 1010 Vienna, Austria Natural History Museum Vienna Vienna Austria

**Keywords:** Distribution, goblin spiders, morphology, new combination, new record, taxonomy

## Abstract

Three new species of *Gamasomorpha* Karsch, 1881 are described: *G. bakeri* Tong & Hörweg, **sp. nov.** (♂♀) (Philippines), *G. fortdekock* Tong & Hörweg, **sp. nov.** (♂) (Indonesia), and *G. jacobsoni* Tong & Hörweg, **sp. nov.** (♂♀) (Indonesia). Additionally, *Xestaspis shoushanensis* Tong & Li, 2014 is recorded for the first time from Sumatra, Indonesia, and transferred to *Gamasomorpha* with a new combination established: *Gamasomorpha shoushanensis* (Tong & Li, 2014), **comb. nov.** Descriptions, diagnoses and photomicroscopy images are provided.

## ﻿Introduction

The family Oonopidae Simon, 1890 comprises tiny spiders, typically measuring between 0.5 and 3 mm in body length. Members of this family have a wide distribution, with particularly high diversity in tropical regions. To date, 1978 extant species assigned to 115 genera are known (WSC 2025).

The genus *Gamasomorpha* Karsch, 1881 currently contains 48 species (WSC 2025) and exhibits a wide distribution, ranging from South America and Africa to Indonesia, China and Australia. This genus is characterized by well-sclerotized scuta, a reddish-brown body color, spineless legs, a palpal bulb that is not fused with the cymbium, and a long, slender embolus that is approximately as long as the bulbus (Eichenberger et al. 2012).

Prior to this study, two species of *Gamasomorpha* had been recorded from the Philippines: *G. cataphracta* Karsch, 1881 and *G. nitida* Simon, 1893. Additionally, two species, *G. kraepelini* Simon, 1905 and *G. seximpressa* Simon, 1907, were described from Java, Indonesia, and one species, *G. semitecta* Simon, 1907, from Sumatra; all were described over a century ago (Simon 1893, 1905, 1907). More recently, seven additional species from Indonesia and adjacent regions have been reported (Eichenberger et al. 2012).

During the examination of oonopid specimens preserved in the Natural History Museum Vienna, Austria, four species from the Philippines and Sumatra, Indonesia were identified. These include three new species of *Gamasomorpha* Karsch, 1881, and the species *Xestaspis shoushanensis* Tong & Li, 2014, which is newly recorded from Sumatra, Indonesia and is transferred to *Gamasomorpha* Karsch herein.

## ﻿Material and methods

The specimens were examined using a Leica M205 C stereomicroscope. Fine details were studied under an Olympus BX51 compound microscope. Endogynes were cleared in lactic acid. Photomicroscope images were taken with a Canon EOS 750D zoom digital camera (24.2 megapixels) mounted on the Olympus BX51. Raw images were initially stacked with Helicon Focus v. 8.2.0 to produce the composite images, which were then processed in Adobe Photoshop CC 2020. Scanning electron microscope images (SEM) were obtained under high vacuum using a Hitachi S-4800 after critical-point drying and gold-palladium coating. The distribution map was generated using ArcGIS v. 10.2 (ESRI Inc.). All measurements were taken with the Olympus BX51 and are given in millimeters. Taxonomic descriptions follow Eichenberger et al. (2012) and Tong and Li (2014). The type material is deposited in the Natural History Museum Vienna, Austria (**NHMW**).

The following abbreviations are used in the text: **ALE** = anterior lateral eyes; **boc** = booklung covers; **ce** = conical extension; **co** = conductor; **cpp** = carapace posterolateral pits; **em** = embolus; **gap** = globular appendix; **kno** = knob; **ma** = mesal embolic accessory appendage; **na** = nail-like process; **PLE** = posterior lateral eyes; **PME** = posterior median eyes; **psc** = paddle-like sclerite; **re** = receptacle; **scr** = scutal ridge; **ssa** = secretory sac; **tg** = transverse groove; **trh** = transparent hairs.

## ﻿Taxonomy

### ﻿Family Oonopidae Simon, 1890

#### ﻿Genus Gamasomorpha

Taxon classificationAnimaliaAraneaeOonopidae

Karsch, 1881

FEB15133-B41C-5245-B11F-3466FE2BC2BC

##### Type species.

*Gamasomorpha cataphracta* Karsch, 1881 from Japan.

##### Comments.

Of the 48 known species in this genus, the geographical distribution is as follows: 19 in Southeast Asia, 9 in East Asia, 4 in South Asia, 3 in West Asia, 9 in Africa, 2 in Australia, and 2 in South America. However, the current classification is problematic. The South American specimens lack illustrations and are likely misidentified. Several African species (e.g., *G. australis* Hewitt, 1915; *G. jeanneli* Fage, 1936; *G. longisetosa* Lawrence, 1952) are evidently misplaced (see fig. 2, Hewitt 1915; fig. 6, Fage and Simon 1936; figs 8–11, Lawrence 1952). Similarly, *G. kabulensis* Roewer, 1960 from Afghanistan may also be incorrectly classified (see fig. 8a–c, Roewer 1960). A comprehensive revision of the genus is therefore essential.

#### ﻿ Gamasomorpha bakeri sp. nov.

Taxon classificationAnimaliaAraneaeOonopidae

DA1C046C-8AEB-5918-9AA5-B84222D73BEA

https://zoobank.org/D97BE1EE-D6EF-4DB5-A0EB-C5603BB3FE49

Figs 1, 2, 3, 12A–C

##### Material examined.

***Holotype*** Philippines • ♂ (NHMW-ZOO-AR-215); Luzon, Mt. Makiling; leg. C.F. Baker. ***Paratypes*.** Philippines • 3♂2♀ (NHMW-ZOO-AR-30391); same data as holotype.

##### Etymology.

The specific name is named after the collector, American entomologist Charles Fuller Baker (1872–1927), who worked in the Philippines from 1899–1903.

##### Diagnosis.

**Figure 1.**
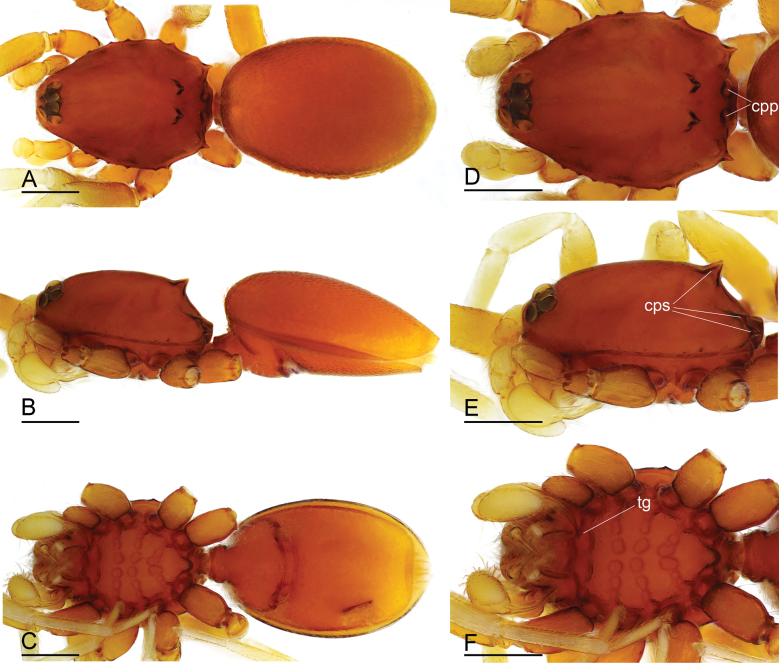
*Gamasomorpha bakeri* sp. nov., male holotype. A–C. Habitus, dorsal, lateral and ventral views; D–F. Prosoma, dorsal, lateral and ventral views. Abbreviations: cpp = carapace posterolateral pits; cps = carapace posterolateral spikes; tg = transverse groove. Scale bars: 0.4 mm.

**Figure 2.**
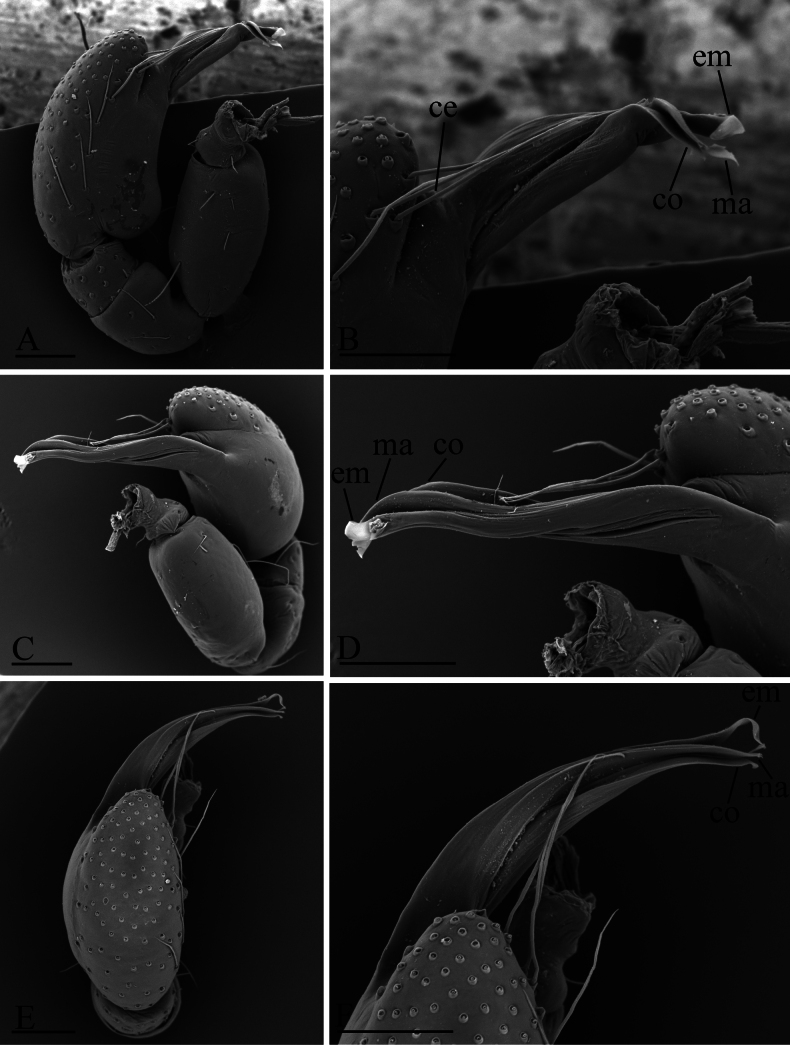
*Gamasomorpha bakeri* sp. nov., male left palp, SEM. A, C, E. Prolateral, anterior-retrolateral and dorsal views; B, D, F. Distal part of bulb, prolateral, anterior-retrolateral and dorsal views. Abbreviations: ce = conical extension; co = conductor; em = embolus; ma = mesal embolic accessory appendage. Scale bars: 0.1 mm.

**Figure 3.**
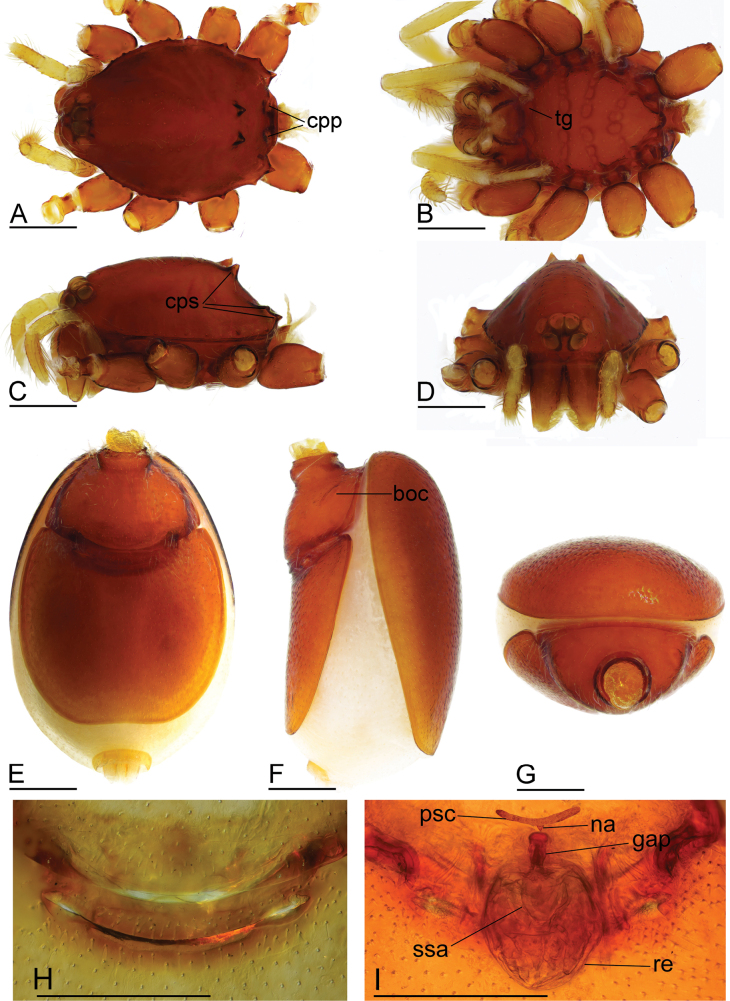
*Gamasomorpha bakeri* sp. nov., female paratype. A–D. Prosoma, dorsal, ventral, lateral and anterior views; E–G. Abdomen, ventral, lateral and anterior views; H. Epigastric region, ventral view; I. Endogyne, dorsal view. Abbreviation: boc = booklung covers; cpp = carapace posterolateral pits; cps = carapace posterolateral spikes; gap = globular appendix; na = nail-like process; psc = paddle-like sclerite; re = receptacle; ssa = secretory sac; tg = transverse groove. Scale bars: 0.4 mm (A–G); 0.1 mm (H, I).

The new species is similar to *G. ophiria* Eichenberger, 2012 from Malaysia in the presence of droplike pits on the sternum, sharply pointed denticles along the lateral margin of the carapace, and posterolateral spikes on the carapace, but can be distinguished by the carapace with three posterolateral spikes (vs two; cf. Fig. 1E and Eichenberger et al. 2012: fig. 15C), the booklung covers are smooth (vs elevated from body surface; cf. Fig. 3F and Eichenberger et al. 2012: fig. 15I, J), and the embolus has a folded tip (vs straight; cf. Fig. 2B, F and Eichenberger et al. 2012: fig. 16A–C).

##### Description.

***Male*** (holotype). Total length 2.78; carapace 1.24 long, 0.91 wide; abdomen 1.49 long, 1.01 wide. Habitus as in Fig. 1A–C. Body reddish brown, legs yellow. Carapace (Fig. 1D, E): surface smooth, with 3 pairs of posterolateral spikes, posterolateral edge with pair of pits; lateral margin with pointed denticles; pars cephalica slightly elevated in lateral view. Eyes (Fig. 1D, E): ALE largest, PLE smallest; posterior eye row straight viewed from above, procurved from front; ALE separated by about their radius; ALE separated from edge of carapace by about 0.8 times their diameter. Sternum (Fig. 1F): longer than wide, surface smooth, with radial furrows of large, roundish, droplike pits between coxae. Abdomen (Fig. 1A–C): dorsal scutum ovoid, punctate, densely covered with short setae; booklung covers middle size; pedicel tube short, without dorsolateral extension; scuto-pedicel region without scutal ridge. Palp (Figs 2A–F, 12A–C): pale-orange; bulb distally tapering, ending as small conical extension (ce); cymbium extending beyond distal tip of bulb; embolus (em) dark, long, slender, lamellar, with folded tip, half way to distal tip split into second, slightly shorter, mesal embolic accessory appendage (ma), adjacent to third, slightly shorter, lamellar conductor (co).

***Female*** (paratype). Total length 2.88; carapace 1.35 long, 0.99 wide; abdomen 1.56 long, 1.21 wide. As in male, except as noted. Epigastric area (Fig. 3E, H): externally without special features. Endogyne (Fig. 3I): receptacle broadly oval, with ovoid secretory sac (ssa), globular appendix (gap) narrow, with anterior paddle-like sclerite (psc) and nail-like process (na), lateral sclerites functioning as muscle attachments.

##### Distribution.

Known only from the type locality (Fig. 13).

#### ﻿ Gamasomorpha fortdekock sp. nov.

Taxon classificationAnimaliaAraneaeOonopidae

49D320C3-1C51-5F97-8FD4-B50BE27FE4CE

https://zoobank.org/0A32030A-BF88-4F4E-BB27-AAE52876B74F

Figs 4, 5, 12D–F

##### Material examined.

***Holotype*** Indonesia • ♂ (NHMW-ZOO-AR-207); Sumatra, Fort de Kock; leg. Edward Richard Jacobson. ***Paratype*.** Indonesia • 2♂ (NHMW-ZOO-AR-30392); same data as holotype.

##### Etymology.

The specific name is a noun in apposition taken from the type locality.

##### Diagnosis.

**Figure 4.**
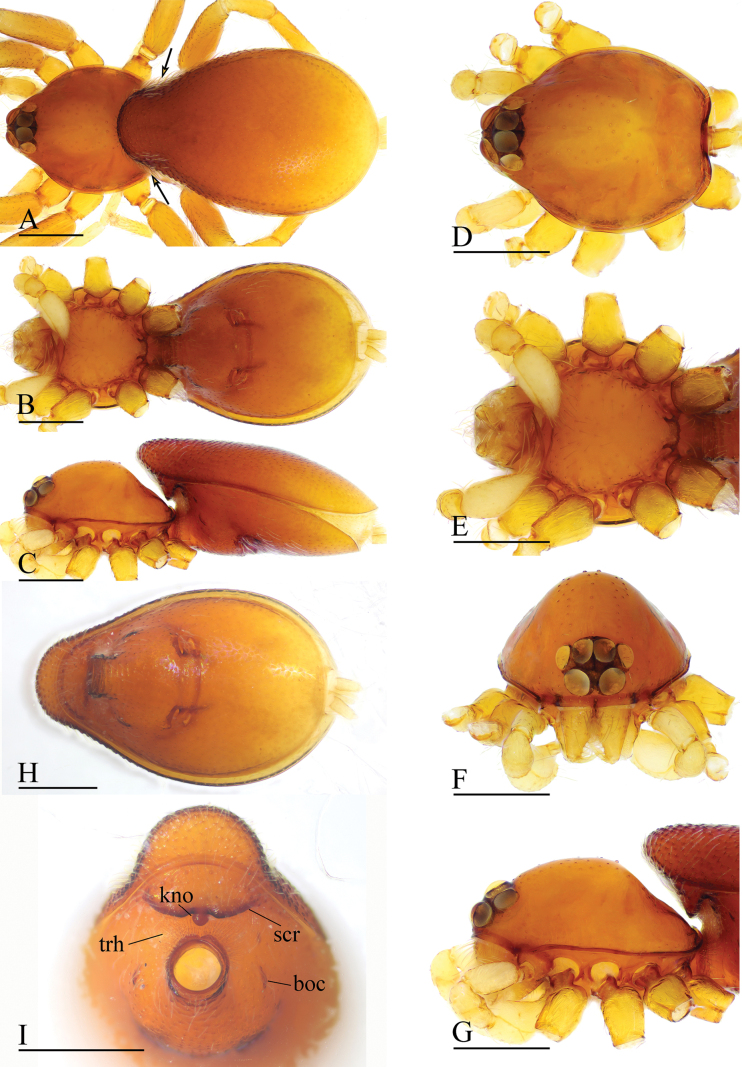
*Gamasomorpha fortdekock* sp. nov., male holotype. A–C. Habitus, dorsal, ventral and lateral views, arrows show the constriction; D–G. Prosoma, dorsal, ventral, anterior and lateral views; H, I. Abdomen, ventral and anteroventral views. Abbreviations: boc = booklung covers; kno = knob; scr = scutal ridge; trh = transparent hairs. Scale bars: 0.4 mm.

The new species is similar to *G. comosa* Tong & Li, 2009 from China in the smooth carapace and the pointed conical extension of bulb, but can be distinguished by the abdomen strongly constricted at anterior part (vs not constrict; cf. Fig. 4A and Tong and Li 2009: fig. 1A), pedicel tube with colorless, almost transparent directed hairs (vs lacking; cf. Fig. 4I and Tong and Li 2009: fig. 2C), and the scuto-pedicel region with a curved scutal ridge and a small round knob (vs lacking, but with nearly rectangular lateral ridge; cf. Fig. 4I and Tong and Li 2009: fig. 2B, C).

##### Description.

**Figure 5.**
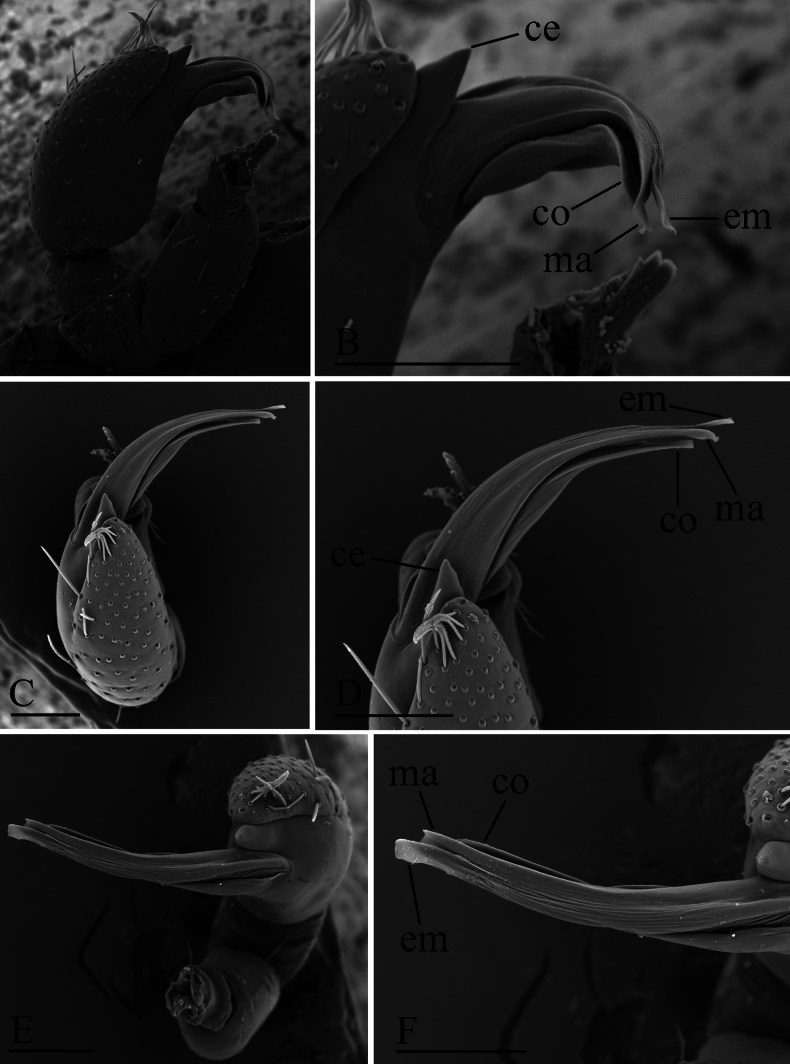
*Gamasomorpha fortdekock* sp. nov., male left palp, SEM. A, C, E. Prolateral, dorsal and anterior-retrolateral views; B, D, F. Distal part of bulb, prolateral, dorsal and anterior-retrolateral views. Abbreviations: ce = conical extension; co = conductor; em = embolus; ma = mesal embolic accessory appendage. Scale bars: 0.1 mm.

***Male*** (holotype). Total length 2.34; carapace 0.95 long, 0.78 wide; abdomen 1.68 long, 1.00 wide. Habitus as in Fig. 4A–C. Body yellow, chelicerae and legs lighter. Carapace (Fig. 4C, D, F): surface smooth; pars cephalica strongly elevated in lateral view; lateral margin with a row of finely hairs. Eyes (Fig. 4D, F): ALE largest, PLE smallest; posterior eye row recurved viewed from above, procurved from front; ALE separated by less than their radius; ALE separated from edge of carapace by about 0.5 times their diameter. Sternum (Fig. 4E): finely reticulate, with narrow, transverse palpal groove, covered with thin hairs standing in small pits, radial furrows present. Abdomen (Fig. 4A, H, I): dorsal scutum ovoid, punctate, densely covered with short setae, anterior part strongly constricted; booklung covers very small; pedicel tube short, without dorsolateral extension, with colorless, almost transparent directed hairs; scuto-pedicel region with curved scutal ridge and a small round knob. Palp (Figs 5A–F, 12D–F): yellowish; bulb distally tapering, ending as pointed conical extension (ce); cymbium not extending beyond distal tip of bulb; with long slender, lamellar embolus (em), adjacent to an embolic accessory appendage (ma) and a lamellar conductor (co).

***Female*.** Unknown.

##### Distribution.

Known only from the type locality (Fig. 13).

#### ﻿ Gamasomorpha jacobsoni sp. nov.

Taxon classificationAnimaliaAraneaeOonopidae

696C6B73-1F25-5538-9C13-541A3D329CB8

https://zoobank.org/9664E26D-58C9-4A1A-BF44-2BB2F28B7625

Figs 6, 7, 8, 12G–I

##### Material examined.

***Holotype*** Indonesia • ♂ (NHMW-ZOO-AR-30393); Sumatra, Fort de Kock; leg. E.R. Jacobson. ***Paratypes*.** Indonesia • 2♂18♀ (NHMW-ZOO-AR-30394); same data as holotype.

##### Etymology.

The specific name is named after the collector, the Dutch entomologist Edward Richard Jacobson (1870–1944).

##### Diagnosis.

**Figure 6.**
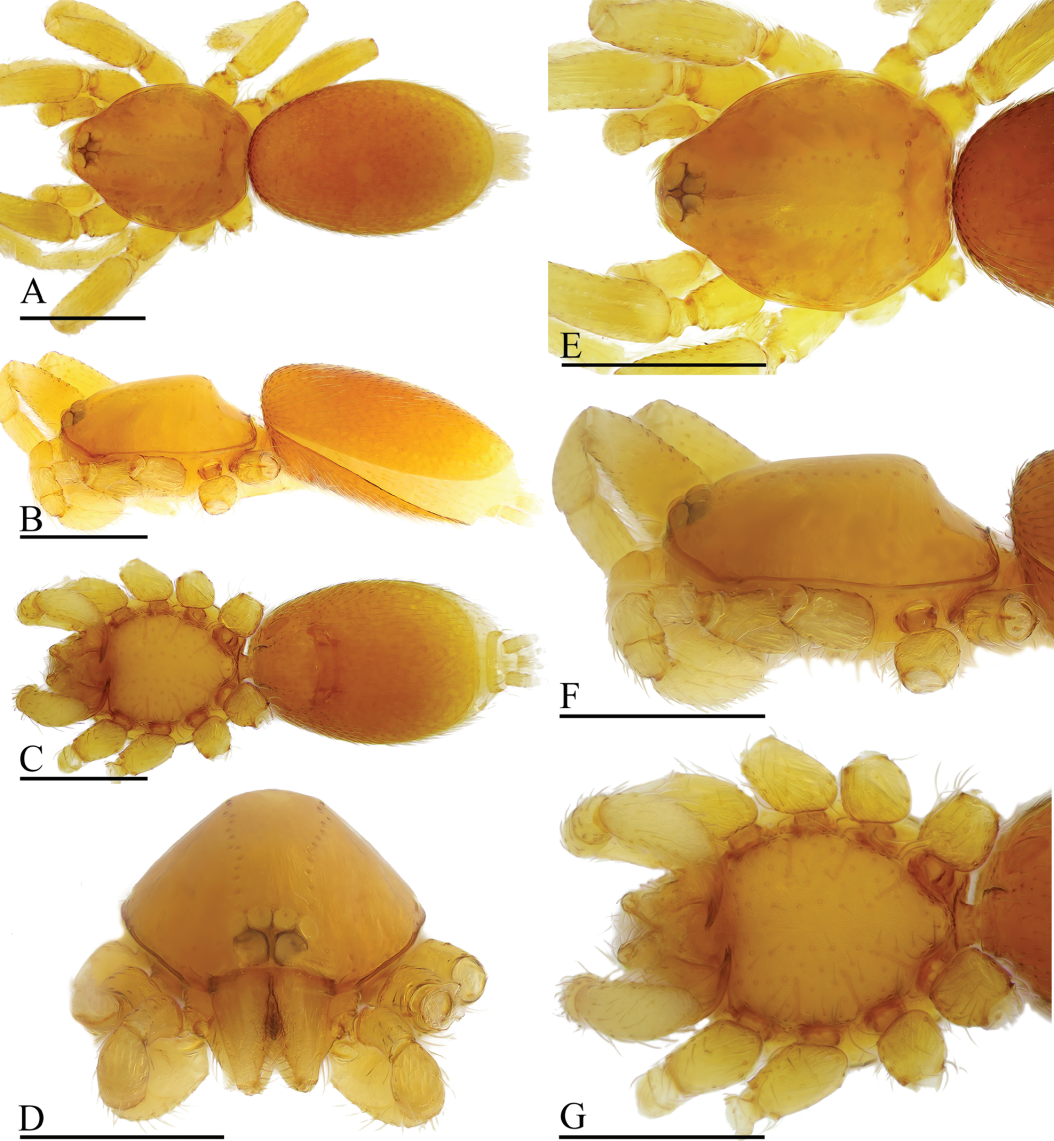
*Gamasomorpha jacobsoni* sp. nov., male holotype. A–C. Habitus, dorsal, lateral and ventral views; D–G. Prosoma, anterior, dorsal, lateral and ventral views. Scale bars: 0.4 mm.

**Figure 7.**
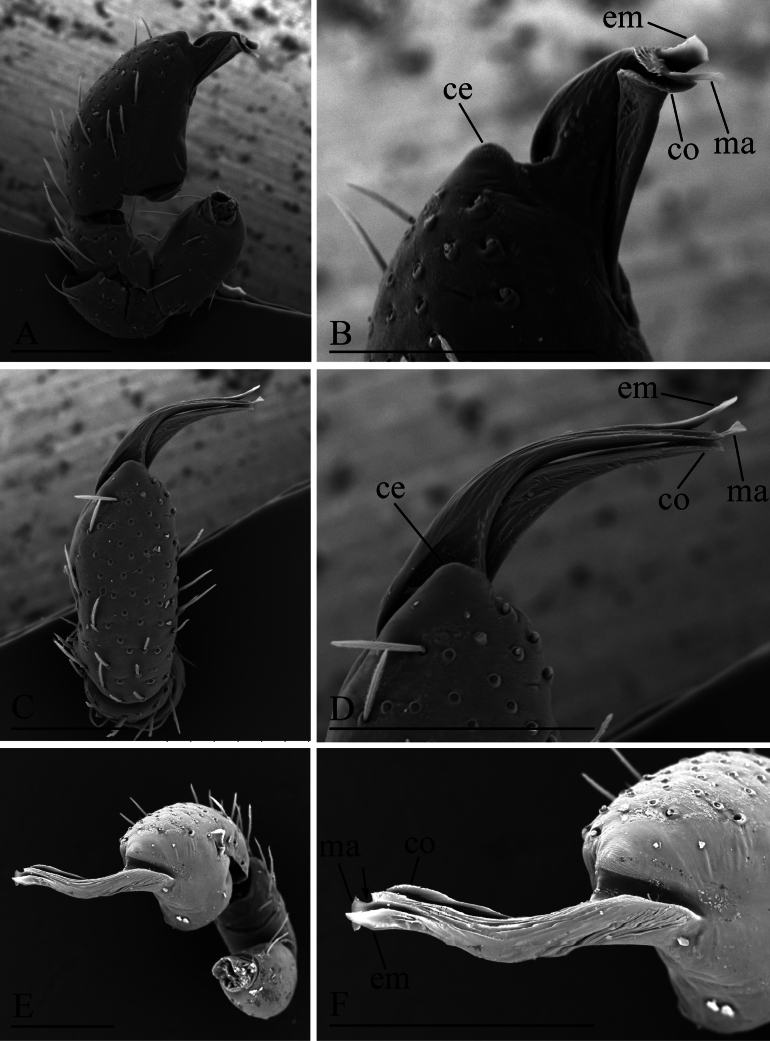
*Gamasomorpha jacobsoni* sp. nov., male left palp, SEM. A, C, E. Prolateral, dorsal and anterior-retrolateral views; B, D, F. Distal part of bulb, prolateral, dorsal and anterior-retrolateral views, arrow shows the subdistal excavation. Abbreviation: ce = conical extension; co = conductor; em = embolus; ma = mesal embolic accessory appendage. Scale bars: 0.1 mm.

**Figure 8.**
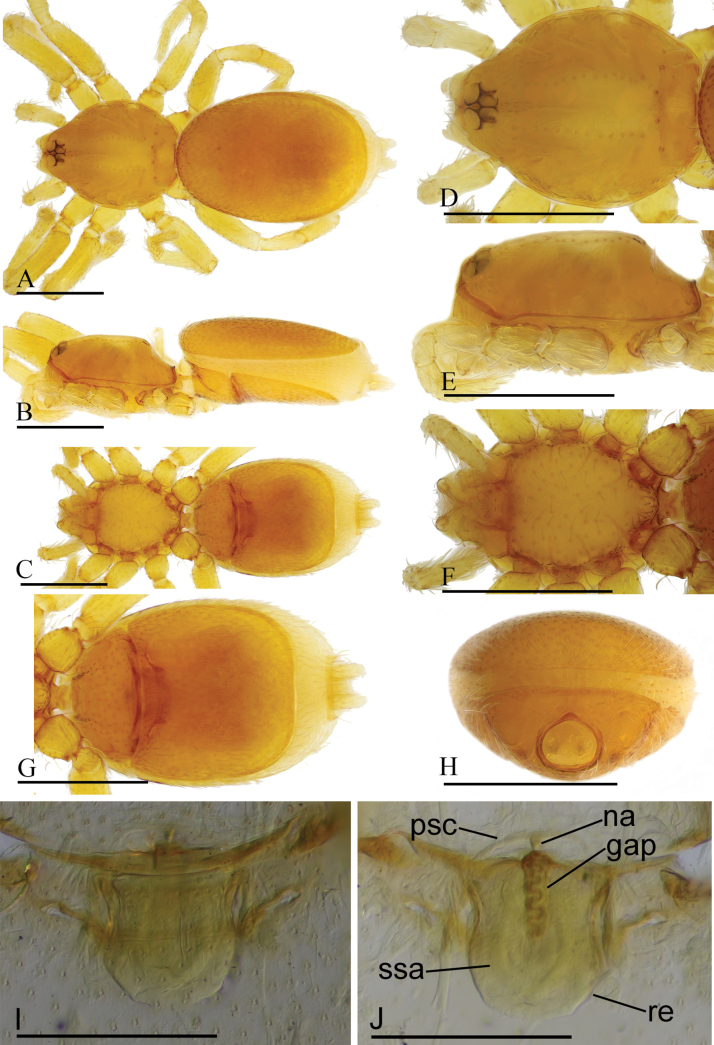
*Gamasomorpha jacobsoni* sp. nov., female paratype. A–C. Habitus, dorsal, lateral and ventral views; D–F. Prosoma, dorsal, lateral and ventral views; G, H. Abdomen, ventral and anterior views; I. Epigastric region, ventral view; J. Endogyne, dorsal view. Abbreviations: gap = globular appendix; na = nail-like process; psc = paddle-like sclerite; re = receptacle; ssa = secretory sac. Scale bars: 0.4 mm (A–H); 0.1 mm (I, J).

The new species is similar to *G. coniacris* Eichenberger, 2012 from Malaysia and Indonesia in the smooth carapace and the broadly-oval receptacle, but can be distinguished by the radial furrow of sternum lacking the droplike pits (vs with droplike pits; cf. Fig. 6G and Eichenberger et al. 2012: fig. 20D), the scuto-pedicel region without scutal ridges (vs with paired curved scutal ridges; cf. Fig. 8H and Eichenberger et al. 2012: fig. 20H), and the bulb with a round conical extension (vs triangular; cf. Figs 7A, 12G and Eichenberger et al. 2012: fig. 21D–F).

##### Description.

***Male*** (holotype). Total length 1.50; carapace 0.59 long, 0.47 wide; abdomen 0.90 long, 0.51 wide. Habitus as in Fig. 6A–C. Body yellow, chelicerae and legs lighter. Carapace (Fig. 6D, E, F): surface smooth; pars cephalica slightly elevated in lateral view. Eyes (Fig. 6D, E): ALE largest, PLE and PME nearly equal size; posterior eye row straight viewed from above, procurved from front; ALE nearly touching; ALE separated from edge of carapace by about 0.6 times their diameter. Sternum (Fig. 6G): smooth, with narrow, transverse palpal groove, radial furrows present. Abdomen (Fig. 6A–C): dorsal scutum ovoid, punctate, densely covered with short setae; booklung covers middle size; pedicel tube short, without dorsolateral extension; scuto-pedicel region without scutal ridge. Palp (Figs 7A–F, 12G–I): pale-orange; bulb distally tapering, ending as round conical extension (ce); cymbium not extending beyond distal tip of bulb; embolus (em) dark, long, slender, lamellar; embolic accessory appendage (ma) with a subdistal excavation; conductor (co) shorter, lamellar.

***Female*** (paratype). Total length 1.66; carapace 0.58 long, 0.48 wide; abdomen 0.96 long, 0.58 wide. Habitus (Fig. 8A–C) as in male, except as noted. Epigastric area (Fig. 8G, I): externally without special features. Endogyne (Fig. 8J): receptacle (re) broadly oval, with ovoid secretory sac (ssa), globular appendix (gap) about half the length of receptacle, with an anterior paddle-like sclerite (psc) and a nail-like process (na), with lateral sclerites functioning as muscle attachments.

##### Distribution.

Known only from the type locality (Fig. 13).

#### ﻿ Gamasomorpha shoushanensis

Taxon classificationAnimaliaAraneaeOonopidae

(Tong & Li, 2014) comb. nov.

1ADEB79F-74C8-50EE-821A-67E2E499EC8D

**Figure 9.**
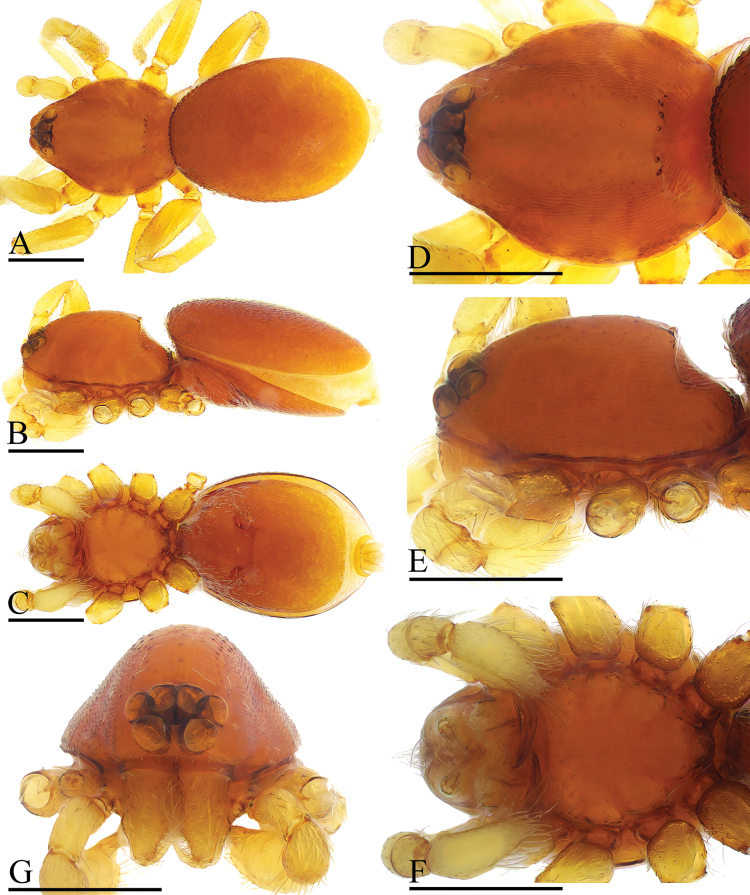
*Gamasomorpha shoushanensis* (Tong & Li, 2014), male. A–C. Habitus, dorsal, lateral and ventral views; D–G. Prosoma, dorsal, lateral, ventral and anterior views. Scale bars: 0.4 mm.

**Figure 10.**
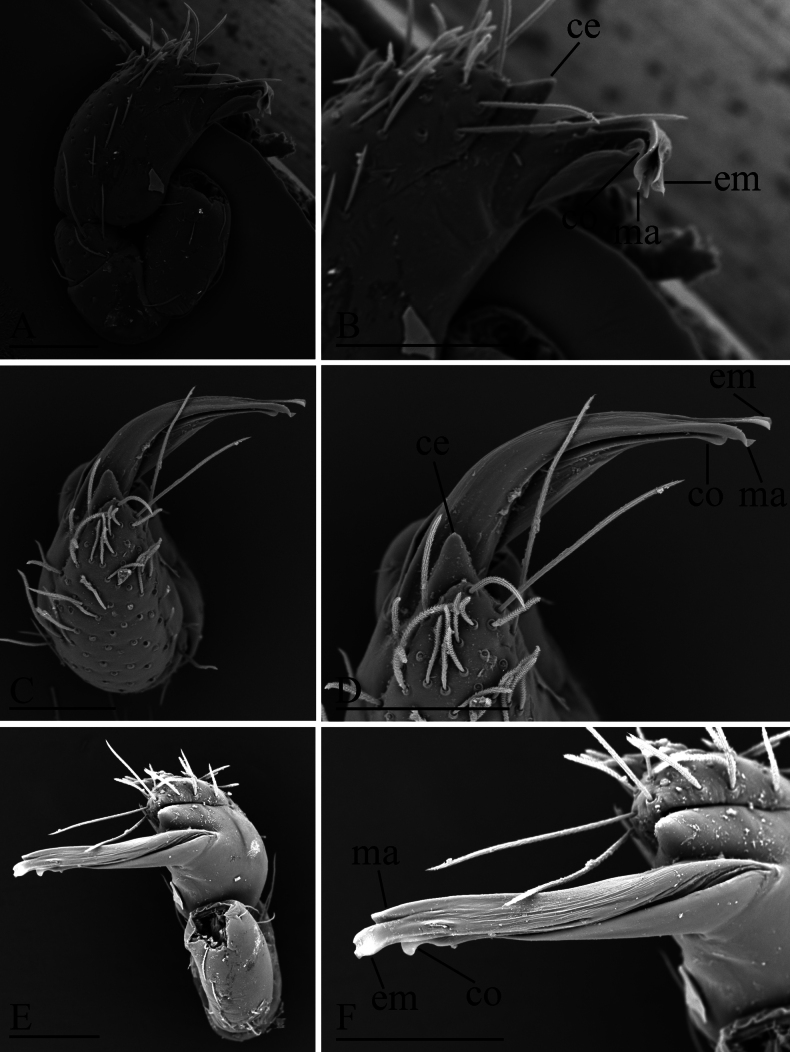
*Gamasomorpha shoushanensis* (Tong & Li, 2014), male left palp. A, C, E. Prolateral, dorsal and anterior-retrolateral views; B, D, F. Distal part of bulb, prolateral, dorsal and anterior-retrolateral views. Abbreviation: ce = conical extension; co = conductor; em = embolus; ma = mesal embolic accessory appendage. Scale bars: 0.1 mm.

**Figure 11.**
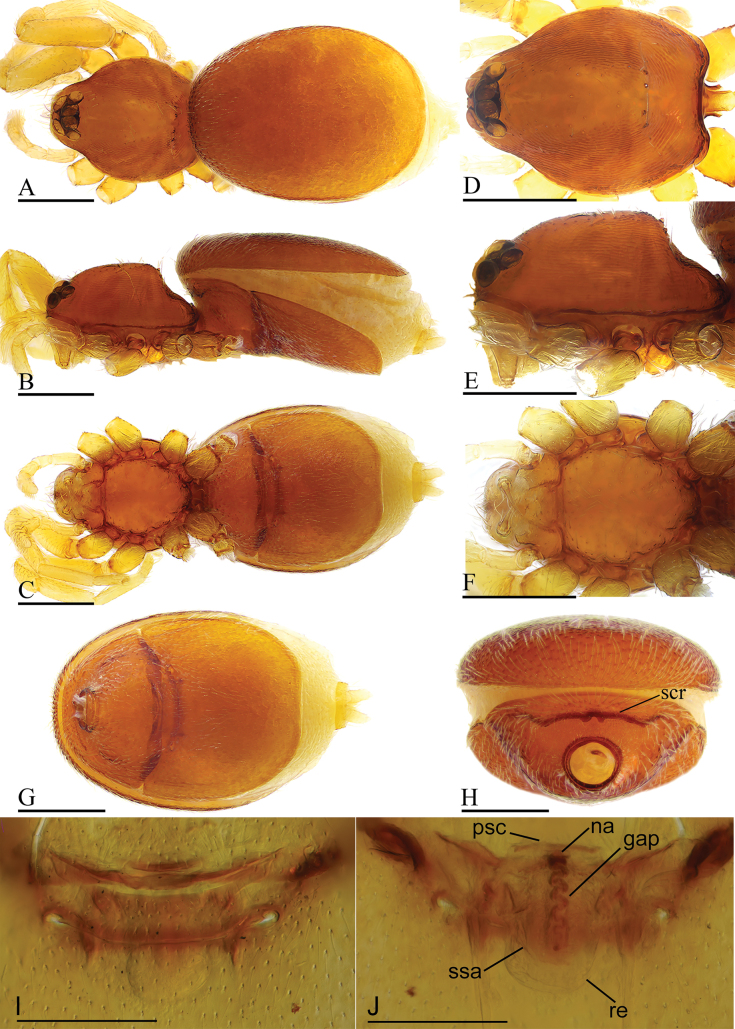
*Gamasomorpha shoushanensis* (Tong & Li, 2014), female. A–C. Habitus, dorsal, lateral and ventral views; D–F. Prosoma, dorsal, lateral and ventral views; G, H. Abdomen, ventral and anterior views; I. Epigastric region, ventral view; J. Endogyne, dorsal view. Abbreviations: gap = globular appendix; na = nail-like process; psc = paddle-like sclerite; re = receptacle; ssa = secretory sac. Scale bars: 0.4 mm (A–H); 0.1 mm (I, J).

**Figure 12.**
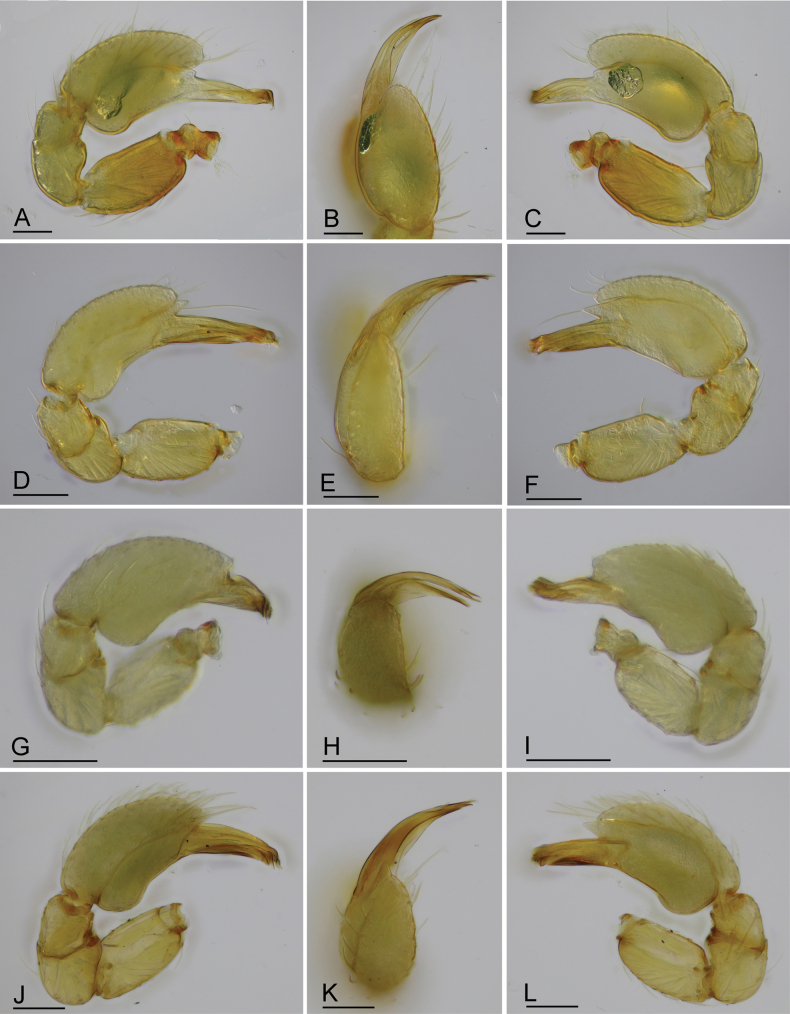
*Gamasomorpha* spp., male left palp. A–C. *G. bakeri* sp. nov.; D–F. *G. fortdekock* sp. nov.; G–I. *G. jacobsoni* sp. nov.; J–L. *G. shoushanensis* (Tong & Li, 2014). A, D, G, J. Prolateral view; B, E, H, K. Dorsal view; C, F, I, L. Retrolateral view. Scale bars: 0.1 mm.

Figs 9, 10, 11, 12J–L

Xestaspis shoushanensis Tong & Li, 2014: 81, figs 8A–K, 9A–J, 10A–C (♂♀).

##### Material examined.

Indonesia • 5♂ 5♀ (NHMW-ZOO-AR-30395); Sumatra, Fort de Kock; leg. E.R. Jacobson. • 13♂ 23♀ (NHMW-ZOO-AR-30396); same data as above.

##### Other materials.

***Holotype*** China • ♂ (IZCAS AR 27810); Taiwan, Kaohsiung City, Shoushan Mt.; 29.VI.2013; S. Li et al. leg. ***Paratypes*.** • 1 ♀ (SYNU-11); same data as holotype • 2 ♀ (SYNU-57); same data as holotype.

##### Diagnosis.

This species is similar to *G. fricki* Eichenberger, 2012 from Vietnam in the striated carapace and the pointed conical extension of bulb, but can be distinguished by the radial furrow of the sternum lacking the droplike pits (vs with droplike pits; cf. Fig. 9F and Eichenberger et al. 2012: fig. 27D), the scuto-pedicel region with a scutal ridge (vs lacking; cf. Fig. 11H and Eichenberger et al. 2012: fig. 28A), and the booklung covers are very small (vs large; cf. Fig. 11G and Eichenberger et al. 2012: fig. 27C).

##### Description.

See Tong and Li (2014).

##### Comments.

Species of *Xestaspis* are similar to those of *Gamasomorpha* except for the presence of a pointed tubercle on the anterolateral face of the epigastric scuta (Ott and Harvey 2008). According to Eichenberger et al. (2012), the separation of the two genera may be arbitrary. The genus *Xestaspis* is most probably a junior synonym of *Gamasomorpha* (Ranasinghe and Benjamin 2016). We re-checked the type specimens of *Xestaspis shoushanensis* and the new materials from Sumatra. The pointed tubercles of the booklung covers of this species are inconspicuous. Thus, we transfer it from *Xestaspis* to *Gamasomorpha*, as a new combination, *Gamasomorpha shoushanensis*.

##### Distribution.

**Figure 13.**
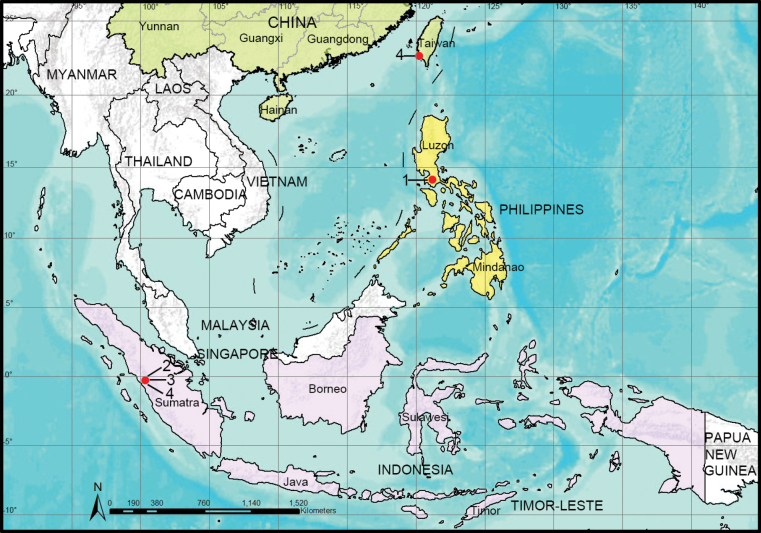
Distribution records of the four *Gamasomorpha* species: 1. *G. bakeri* sp. nov.; 2. *G. fortdekock* sp. nov.; 3. *G. jacobsoni* sp. nov.; 4. *G. shoushanensis* (Tong & Li, 2014).

China (Taiwan), Indonesia (Fig. 13).

## Supplementary Material

XML Treatment for Gamasomorpha

XML Treatment for Gamasomorpha bakeri

XML Treatment for Gamasomorpha fortdekock

XML Treatment for Gamasomorpha jacobsoni

XML Treatment for Gamasomorpha shoushanensis

## ﻿Additional information

### Use of AI

No use of AI was reported.

### Author ORCIDs

Yang Bai https://orcid.org/0009-0004-0346-8685

Dongju Bian https://orcid.org/0000-0002-9324-8026

Yanfeng Tong https://orcid.org/0000-0002-4348-7029

Christoph Hörweg https://orcid.org/0000-0002-8208-0758

### Data availability

All of the data that support the findings of this study are available in the main text.
